# Treatment patterns of childhood diarrhoea in rural Uganda: a cross-sectional survey

**DOI:** 10.1186/1472-698X-12-19

**Published:** 2012-09-25

**Authors:** Jenny Löfgren, Wenjing Tao, Elin Larsson, Francis Kyakulaga, Birger C Forsberg

**Affiliations:** 1 Division of Global Health, Department of Public Health Sciences, Karolinska Institutet, Nobels väg 9, 171 77, Stockholm, Sweden; 2 Department of Health Sciences, Uganda Christian University, Mukono, Uganda

**Keywords:** Diarrhoea, Diarrhoea case management, Diarrhoea control, Oral rehydration, Child health, Uganda

## Abstract

**Background:**

Diarrhoea is the second leading cause of death in children under five accounting for 1.8 million deaths yearly. Despite global efforts to reduce diarrhoea mortality through promotion of proper case management, there is still room for ample improvement. In order to seek options for such improvements this study explored the knowledge and practices of diarrhoea case management among health care providers at health centres and drug shops in Uganda.

**Methods:**

Records were reviewed for case management and structured interviews concerning knowledge and practices were conducted with the staff at all health centres and at all identified drug shops in the rural district of Namutumba, Uganda.

**Results:**

There was a significant gap between knowledge and documented practices among staff. Antibiotics, antimalarials and antipyretics were prescribed or recommended as frequently as Oral Rehydration Solution (ORS). In almost a third of the health facilities, ORS was out of stock. 81% of staff in health centres and 87% of staff in drug shops stated that they prescribed antibiotics for common diarrhoea. Zinc was not prescribed or recommended in any case.

**Conclusions:**

The findings indicate that many children presenting with diarrhoea are inadequately treated. As a result they may not get the rehydration they need and are at risk of potential side effects from unjustified usage of antibiotics. Practices must be improved at health centres and drug shops in order to reduce childhood mortality due to diarrhoeal diseases.

## Background

Diarrhoea is the second leading cause of under five mortality globally and it accounts for 1.8 million deaths annually [1]. The majority of these deaths occur in south Asia and Africa [2]. According to Bhutta et al., childhood mortality has fallen by 28% since 1990 in 68 prioritized countries. However, efforts need to increase substantially to achieve Millennium Development Goal 4 (MDG4) of reducing childhood deaths [3].

In order to reduce mortality and morbidity due to childhood diarrhoea, the “Programme for Control of Diarrhoeal Diseases” (CDD) was initiated by WHO at the beginning of the 1980s [4]. Diarrhoea caused an estimated 4.6 million deaths in children under five at that time [5]. The major cause of death due to diarrhoeal diseases is dehydration. The cornerstone in diarrhoea case management is to prevent or treat dehydration [6]. Oral Rehydration Therapy (ORT) was therefore chosen as the primary intervention in the CDD programme. Subsequently, usage of ORT increased which contributed to reductions in diarrhoea mortality in low- and middle-income countries (LMIC) [7,8]. Even so, the usage of ORT is still unsatisfactory in many LMIC [9].

In 2004, an additional therapy, zinc, was added to the recommended treatment of diarrhoea in children. Zinc has been found to reduce the duration and severity of the diarrhoea episode and lower the incidence of diarrhoea during the months following the episode [10-12]. Later, zinc has also been shown to reduce mortality from diarrhea [13].

Advice on health issues in low-income countries is often sought from private practitioners and drug vendors [14,15]. In many cases, an untrained drug vendor becomes an important source of information and treatment during childhood diarrhoea [16].

This study investigated knowledge and practices among staff at health centres (HC) and drug shops (DS) in a rural setting in Uganda in order to explore the scope for improvement of diarrhoea case management.

## Methods

### Study setting

The study was carried out in Namutumba district, located about 125 km north east of Kampala, the capital of Uganda. This rural district had a population close to 200 000 at the time of the study. There were 33 registered HC in the district (21 governmental, 12 NGO run). Health centres in Namutumba district range from level two to level four. (Health facilities in Uganda are divided into seven levels where level 1 is the village health team, 2–4 HC, 5–6 hospitals and 7 the national referral hospital in Kampala.) There were no pharmacies in the district while 10 drug shops were registered with the district headquarters.

### Study participants and data collection

All registered health centres in Namutumba district were included in the study. In each facility a structured interview was carried out by the lead researcher assisted by an interpreter. In the lower level health centres there was typically only one staff available. When more than one staff was present the best educated person was interviewed. All staff approached consented to participate. In the interviews respondents’ knowledge on diarrhoea and diarrhoea management as well as their stated practices were assessed. The questionnaire was constructed with a combination of closed and open ended questions.

A review of the outpatient register was also done by the lead researcher. Information about diagnosis and recommended treatment for all cases of diarrhoea in children under the age of five during the past two months were recorded. A facility inventory was made in all facilities to check for availability of ORS, drugs and clinical guidelines.

Interviews, record reviews and inventories were conducted in all but two HC that were closed at the time of visit.

Drug shops were identified on arrival in the 24 villages where the HC were located. All drug shops that could be identified were visited by the research team. In most (n = 46) of the identified DS, a structured interview was performed by the lead researcher with the assistance of an interpreter. In most cases there was only one person attending the DS. The design of the questionnaire was similar to the one used in the HC. In four cases, interviews could not be undertaken because the staff either declined to participate or was absent.

### Data analysis

Data were entered in MS Excel 2011, cleaned for any inconsistencies and analysed for standard distribution measurements. Student’s two-tailed t-tests were performed to determine p-values for differences between health centres and drug shops. P-value < 0.05 was considered significant. Calculations of confidence intervals assumed normal distribution of proportions.

#### Ethics

The study was approved by the Ethics Committee at Uganda Christian University, Mukono, Uganda. All participants gave informed consent and signed a consent form prior to participation in the study.

## Results

77% (24) of the HC visited were level two facilities, 19% (6) level three and 3% (1) level four. The respondents in the HC were nursing assistants (65%), nurses/midwives (26%) or clinical officers (15%). All respondents in both HC and DS had 3–6 years of secondary schooling and a majority of the respondents at the HC had attended a nursing school. The respondents at the DS were nursing assistants (82%) or nurses/midwives (15%). One person was a veterinary assistant (2%).

### Knowledge about causes, symptoms and danger signs of diarrhoea in children

The respondents were asked to mention causes, symptoms and danger signs of childhood diarrhoea. The causes have been classified into four categories: feeding and drinking, hygiene, microbes, and concomitant diseases (table 1). The symptoms and danger signs are divided into four categories: dehydration, gastrointestinal, general condition and other. The three most commonly stated symptoms in the respective category are listed in table 2. (Respondents gave multiple answers.)

**Table 1:** Causes of diarrhoea as stated by respondents at health centres and drug shops

	**Health centre (n = 31)**	**Drug shop (n = 46)**
**Feeding and drinking**	**24 (77%)**	**35 (76%)**
Contaminated food and feeding practices	20 (65%)	31 (67%)
Contaminated water	13 (42%)	26 (57%)
Malnutrition	8 (26%)	2 (4%)
Weaning/mixing food	5 (16%)	2 (4%)
**Hygiene, sanitation and environment**	**19 (61%)**	**24 (52%)**
Microbes	16 (52%)	8 (17%)
Bacteria	6 (19%)	2 (9%)
Worms	15 (48%)	5 (11%)
Other	3 (16%)	-
**Other diseases**	**20 (65%)**	**25 (54%)**
Malaria	20 (65%)	25 (54%)
Measles	7 (23%)	10 (22%)
Other	4 (13%)	3 (7%)
**Other causes**	**5 (16%)**	**2 (7%)**

**Table 2:** Symptoms and danger signs of diarrhoea as stated by the respondents at health centres and drug shops (%)

	**Regular symptoms**		**Danger signs**	
	**Health centre (n = 31)**	**Drug shop (n = 46)**	**Health centre (n = 31)**	**Drug shop (n = 46)**
**Dehydration symptoms**	**68**	**30**	**77**	**43**
Sunken eyes	26	15	48	35
Decreased skin turgor	52	13	26	26
Thirst	26	4	13	2
**Gastrointestinal symptoms**	**48**	**33**	**16**	**24**
Loose and frequent stools	35	7	10	4
Abdominal discomfort and pain	13	13	-	2
Vomiting	10	7	6	4
**General condition**	**61**	**70**	**45**	**41**
Weakness and malaise	32	41	26	22
Loss of apetite	19	33	10	9
Altered body temperature	16	7	3	15
**Other symptoms**	**19**	**20**	**13**	**11**
**Do not know**	**-**	**7**	**-**	**-**

There was a statistically significant difference (P < 0.05) between the respondents at the HC and DS regarding knowledge about causes (p = 0.03), but not about symptoms (p = 0.12) and danger signs (p = 0.33). The respondents in the HC more frequently (52%) mentioned microbes as pathogens in diarrhoea than those in the DS (17%). Staff in the HC was more familiar with signs of dehydration both as symptom (68% vs. 30%) and danger sign (77% vs. 43%) than the staff in DS. At the HC, the frequency of correct and detailed answers increased with the level of training. For example, 100% of the clinical officers and nurses mentioned dehydration symptoms while only 75% of the nursing assistants did so. All clinical officers mentioned several microbes as causing diarrhea compared to 75% and 35% of the nurses and nursing assistants respectively.

### Practices in childhood diarrhoea case management

A majority of the respondents at the HC (87%) and the DS (67%) mentioned ORS as the primary treatment in childhood diarrhoea, followed by antibiotics (13% at HC, 26% at DS). The most desired stated outcome from treating diarrhoea was cure or reduction of the frequency of loose stools (42% in HC, 70% in DS). The second most wanted outcome was rehydration (39% in the HC, 20% in the DS). In the HC, better educated staff was more prone to mention rehydration as the desired outcome than less educated staff.

When asked about actual practices, 87% (HC) and 70% (DS) mentioned that they gave ORS. 81% (HC) and 87% (DS) stated that they prescribed antibiotics. Antimotility drugs were never mentioned in the HC, but by 9% of the respondents in DS. Zinc was never mentioned as a recommended treatment, neither in HC nor in DS, but 55% at the HC and 50% at the DS had heard about the zinc preparation promoted in Uganda. At the time of the study, zinc was not included in the clinical guidelines from the Ministry of Health (MoH) and it was not provided to the health centres. Differences in practices between HC and DS were not statistically significant.

The facility inventory showed that 77% of the HC and 63% of the DS had ORS in stock. The stated reason for stock outs at the HC was delayed delivery from central medicine depots, and in the DS that the owner had not ordered ORS. MOH clinical guidelines were available in 68% of the HC and in 4% of the DS.

### Record review

In the record review, 523 cases of childhood diarrhoea were identified. Most cases were diagnosed and treated for concomitant conditions such as malaria (83%, 95% Confidence Interval (CI) 80–86) and respiratory tract infection (16%, 95% CI 13–19). Dysentery was uncommon (2%, 95% CI 1–3). The most commonly recommended treatments were antimalarials (82%, 95% CI 79–85) and analgetics (80%, 95% CI 77–83) followed by rehydration therapy (78%, 95% CI 74–82), antibiotics (76%, 95% CI 72–80), deworming medicines (32%, 95% CI 29–35), vitamins and trace elements (16%, 95% CI 13–19) and other (4%, 95% CI 2–6).

## Discussion

Diarrhoeal diseases is a major target in the global efforts to increase survival and reduce the disease burden in children [2]. Prevention is of primary importance in these efforts but adequate management of cases is essential to reduce mortality, in particular the administration of Oral Rehydration Solutions (ORS) to treat dehydration [3]. The findings in this study highlight areas for improvement in diarrhoea case management.

Respondents’ knowledge about symptoms and danger signs of dehydration showed positive results but also that there was room for improvement. Such knowledge is essential as most deaths from diarrhoea are caused by dehydration. Staff in HC generally scored better than those in DS. Further training of staff on diarrhoea, its symptoms and potential consequences is called for. In particular, clinic based training with hands-on experience from treating cases should be considered as it has proven to be particularly effective [4].

Knowledge on treatment with ORS was generally good. ORS was the stated first hand treatment choice in 87% of the interviews in the HC and in 67% of the DS. Equally many (87% respectively 70%) claimed that they recommend ORS in real practice. In the record review 78% of the cases had been given rehydration therapy. Still, it was only the third most commonly recommended medicine in childhood diarrhoea. In one third of the facilities ORS was out of stock.

Overuse of inappropriate medication during childhood diarrhoea is common [15,16]. Antibiotics are extensively used in diarrhoea case management [17-19], even though they are only recommended in few cases of diarrhoea. Anti-diarrhoeal medicines are not recommended at all in children [20-22].

Antibiotics are often attained from other sources than from trained medical personnel [23]. In the present study over 80% of the respondents stated they recommend antibiotics. Still, antibiotics are only indicated in dysentery, cholera and for certain cases of persistent diarrhea [2,24]. Overuse cause potentially harmful side effects and contributes to bacterial resistance development [25]. In the record review, dysentery was a rare diagnosis (2%) and it cannot motivate that 76% of the cases were recommended antibiotics.

Despite the high use of antibiotics, microbes were not mentioned equally frequent as pathogens in diarrhoea (52% in HC and 17% in DS). Worms and bacteria were the most well-known microbes while viral pathogens, that often cause childhood diarrhea [26], were never mentioned. Improved knowledge about diarrhoea etiology and when to treat with antibiotics, could possibly decrease the antibiotic usage.

Antidiarrhoeals were never recommended in the HC and sold in only 9% of the DS, corresponding to a much lower use rate than that of previous studies [21].

Previous studies have suggested that caretakers may prefer a combination of ORS and other medicines to single therapy with ORS. Zinc has shown to increase ORS usage [27] and reduce the use of antibiotics and antidiarrhoeals [28]. In our study zinc was never used but half of the respondents had heard about the medicine. A recent study from India showed that, even when available, zinc is rarely used [29]. Zinc was introduced as standard treatment in childhood diarrhoea through the WHO/UNICEF joint statement on the clinical management of acute diarrhoea in 2004 [10]. However, it is often unavailable in most countries due to difficulties in including it in national policy plans or due to lack of funding [30]. This was also the case in Uganda at the time of the study. Efforts need to be made to increase the availability of zinc so that children with diarrhoea can benefit from the therapy.

Correct management of diseases relies on correct knowledge on causes, symptoms and therapies. The frequency of correct answers in the interviews increased with the level of education, but care was often provided by the least educated personnel. Adequate and continuous training will most likely contribute to improved diarrhoea case management. Training within the IMCI strategy has previously shown to significantly improve the quality of care for children under five [31].

Drug shops are often the first line of health care in low-income settings like Namutumba district so it is crucial to also involve drug shop attendants in training. Registration and monitoring of DS is needed to reach this group of health care providers with information and training opportunities.

We agree with other researchers that “training is not enough” [31] and other measures must be taken parallel to knowledge transfer to achieve desired changes in behaviour. Still knowledge among providers is a necessary pre-requisite for large-scale and sustainable improvements in case management.

Most children in the record review were recommended treatment for concomitant diseases. Possibly the integrated child case management has contributed to this diagnostic overlap as diagnoses in this setting generally are based on history and clinical examination. In reality, cross diagnosing decrease the risk of missing a case of any of the three potentially life-threatening childhood diseases in the study setting (malaria, pneumonia and diarrhoea). The issue of overlapping symptoms in child illnesses in Uganda has been extensively studied and discussed for pneumonia and malaria [32]. Overlap issues in diarrhoea have been much less studied and should be a focus in future research.

### Methodological considerations

The results from this provider survey are higher for ORS prescription than for ORS use as documented in community household surveys from the area. There are several reasons for this discrepancy. One is that some community members do not seek care from the providers investigated and their case management is likely to differ from those who seek a provider′s advice. Another is that the ORS prescribed may not be bought or little, if at all, used. Hence, we cannot draw conclusions on community practices from a survey of providers.

The record reviews at the HC registered the recommended medicines. It was not specified whether the medicines were given at the facility or had to be purchased elsewhere.

Most drug shops in the district were visited but since there was no official registration of DS, some DS may have been missed in the sample. However, it was clear from interviews that the large majority of drug shops were identified and included in the study.

The study was carried out in a rural district in Uganda which limits its generalisability. However, as documented its findings are in line with findings from other places.

The principal investigator of the study was performing the interviews together with an interpreter. This arrangement was selected to improve quality control. However, it may have increased the risk of over-reporting good practices. Generally, self-reported practices have lower validity than other more objective measurements. In this study, record reviews served as such a measurement. It is a limitation of the study that no such records were available in drug shops.

## Conclusions

The findings show that ORS use is well established but antibiotics continue to be overprescribed and the zinc usage is not taking off as it should. A number of interventions are needed to improve the service delivery. Continuous training for health care providers in both HC and DS is a necessary, though not always sufficient, pre-condition for improved practices. Particular efforts should be made to increase ORS usage, to introduce zinc in routine practice and to decrease the significant overuse of antibiotics. Procurement and logistics around delivery of medicines need to be improved so that ORS and zinc are always in stock in HC and DS. The study underlines the need for intensified operational research on implementation of recommended diarrhoea case management as proposed by an international study group in 2009 [33]. Such research is even more called for after the recent appeal by President Museveni and former US President Bill Clinton to accelerate the fight against diarrhoea in Uganda [34].

## Competing interests

The authors declare that they have no competing interests.

## Authors’ contributions

JL designed the study along with co-authors, performed the data collection, analysed data and wrote the manuscript. WT participated in the data collection and study design. EL contributed to the study design and data analysis. FK supervised the data collection and contributed to the study design. BF initiated the study and supervised all parts of it, including study design, data analysis and manuscript editing. All authors commented on the manuscript. All authors read and approved the final manuscript.

## Pre-publication history

The pre-publication history for this paper can be accessed here:

http://www.biomedcentral.com/1472-698X/12/19/prepub
